# Updates in Biliary Tract Cancers

**DOI:** 10.3390/cancers14112746

**Published:** 2022-06-01

**Authors:** Daneng Li, Ya-Han Zhang, Christiana J. Crook, Renuka V. Iyer

**Affiliations:** 1 Department of Medical Oncology and Therapeutics Research, City of Hope, Duarte, CA 91010, USA; yahanzhang01@gmail.com (Y.-H.Z.); ccrook@coh.org (C.J.C.); 2 Roswell Park Cancer Institute, Buffalo, NY 14263, USA; renuka.iyer@roswellpark.org

Biliary tract cancers (BTCs) are a heterogeneous group of malignancies arising from the epithelium of the biliary tree. They include cholangiocarcinoma (CCA) (intrahepatic, perihilar, and distal) and gallbladder cancer (GBC) [1,2]. Although BTCs are a rare diagnosis, accounting for approximately 3% of all adult cancer diagnoses [2], their incidence is rising [3]. This Special Issue offers valuable insights into risk factors, management, and treatment of patients with BTCs through a series of ten papers (five original research articles and five reviews) presented by international leaders in the field.

The treatment options for BTCs are limited; the only curative option is surgical resection [1]. Unfortunately, many patients present with unresectable or metastatic disease, at which point systemic therapy is often the only available treatment modality [1,4]. Cowzer and Harding provide a review of the current and emerging treatment landscape for patients with BTC [5]. While a combination of gemcitabine and cisplatin has been the standard first-line treatment for many years, the number of available treatments has recently expanded to include additional chemotherapy regimens, targeted precision treatments, and immunotherapy drugs that are given alone or in combination with chemotherapy. Many ongoing clinical trials are likely to further expand the systemic treatment arsenal for BTC patients.

The treatment advances described by Cowzer and Harding are impressive, yet the fact remains that these treatments are given to patients with advanced BTC as a palliative measure. There is a current lack of knowledge regarding potential risk and prognostic factors associated with a BTC diagnosis. To date, hepatitis B and C, alcohol, and environmental toxins are known risk factors for intrahepatic CCA. De Lorenzo et al. recently identified non-alcoholic steatohepatitis (NASH) as a novel risk factor for intrahepatic CCA [6]. Patients with NASH-related intrahepatic CCA had shorter overall survival than intrahepatic CCA patients without classic risk factors. In GBC patients, Giannis et al. found that age ≥70 years, higher Charlson–Deyo score, non-private insurance, and higher tumor grade were associated with worse prognosis [7]. Additionally, they compared the prognostic ability of the 8th and 7th editions of the American Joint Commission on Cancer (AJCC) GBC staging and found that overall survival concordance indices were similar for patients classified with the 8th edition versus the 7th edition. Changes in the 8th edition N classification did not improve the prognostic performance of this staging system, but the risk of death appeared to be higher for N2 versus N1 patients of the same T stage.

Managing the disease complications of BTC patients can be difficult. BTC patients typically suffer from poor quality of life due to both tumor- and treatment-related complications. As part of this Special Issue, Hunter and Soares provide a review of common complications BTC patients experience and their effects on patients’ quality of life [8]. They note that BTC patients often develop obstructive complications, which are typically resolved with biliary stent placement. However, stent placement increases the risk of developing cholangitis. Accompanying this review is an article by Iyer et al. on the management of emergency symptoms in stented CCA patients [9]. A RAND/UCLA modified Delphi panel of experts agreed that white blood cell count, bilirubin level, and fever were key drivers in deciding between stent manipulation, inpatient antibiotics, or outpatient antibiotics for patients with stents requiring emergency treatment. Stent manipulation is recommended in patients with elevated bilirubin or patients with normal bilirubin who present with new or worsening biliary dilation. Inpatient antibiotic intervention is appropriate in febrile patients, neutropenic patients, patients with elevated white blood cell count, afebrile patients with elevated bilirubin, and afebrile patients with normal bilirubin who present with new or worsening biliary dilation.

As the treatment landscape for BTCs continues to evolve, an area of heightened interest has been the role of genomic profiling in identifying potential targeted therapy approaches. In a study of 114 patients with CCA who received surgical resection with curative intent between 2010 and 2020, Thornblade et al. found that 36 patients (32%) had undergone tumor genomic profiling [10]. There was a median of two actionable mutations per patient, and a median of one drug per patient was associated with the identified mutation(s). Common mutations included *TP53*, *KRAS*, *IDH1/2*, *BRAF*, and *PIK3CA*. The authors note that while tumor genomic profiling has become more common, general knowledge and perceived utility of targeted therapies may still be low among providers. Thus, several articles in this Special Issue highlight recent advancements in the identification of genomic alterations and the role of precision medicine in the treatment of BTC patients.

The standard first-line treatment for BTC patients is a combination of gemcitabine and cisplatin, but CCA is known for its poor response to chemotherapy. Marin et al. provide a review of mechanisms of pharmacoresistance which limit the efficacy of first-line gemcitabine and cisplatin therapy. They also include valuable insights into the molecular mechanisms of targeted therapies and immunotherapeutic agents used to treat BTC [11]. Among targeted therapies, fibroblast growth factor receptor (FGFR) inhibitors have emerged as a viable treatment option for advanced and metastatic CCA. This Special Issue features a review by Lee et al. of CCA genetic features, the biology of the FGFR pathway in advanced CCA, and important FGFR inhibitor clinical trials [12]. In addition to FGFR inhibitors, Yin et al. provide a review of poly (ADP-ribose) polymerase (PARP) inhibitors and their role in blocking DNA damage repair pathways [13]. The authors discuss genetic mutations that are potentially targetable by PARP inhibitors in BTC, including *ATM*, *IDH1*, and *RAD51/52*, and present an anecdotal case of a patient with BTC who benefited from combination PARP inhibition and immune checkpoint inhibitor therapy. Other researchers aim to shed more light on human epidermal growth factor receptor 2 (HER2)–targeted therapy in advanced BTC. In their pilot study, Jeong et al. show that a combination of trastuzumab-pkrb, gemcitabine, and cisplatin has promising preliminary feasibility in treating patients with HER2-positive advanced BTC [14]. With the approval of two FGFR inhibitors for targeted treatment of CCA [15,16], a number of clinical trials investigating the role of PARP inhibition in BTC [17], and a promising response to combination HER2-targeting with pertuzumab plus trastuzumab in advanced BTC patients [18], Lee et al., Yin et al., and Jeong et al.’s discussions of resistance to FGFR inhibitors, the potential of PARP inhibition, and combination HER2 biosimilar plus gemcitabine plus cisplatin are very timely for clinicians. 

The wide range of topics discussed in this collection of articles highlights the rapid growth of knowledge in the diagnosis, treatment, and management of BTC. We hope these articles will help researchers expand their knowledge of BTCs and guide them in the development of optimal treatment management plans for this rare patient population.
